# Correction: Border-associated macrophages promote cerebral amyloid angiopathy and cognitive impairment through vascular oxidative stress

**DOI:** 10.1186/s13024-024-00743-7

**Published:** 2024-07-12

**Authors:** Ken Uekawa, Yorito Hattori, Sung Ji Ahn, James Seo, Nicole Casey, Antoine Anfray, Ping Zhou, Wenjie Luo, Josef Anrather, Laibaik Park, Costantino Iadecola

**Affiliations:** Feil Family Brain and Mind Research Institute, Weill Cornell Medicine, New York, NY 10021 USA

**Correction: Molecular Neurodegeneration (2023) 18:73**

10.1186/s13024-023-00660-1

The original article contains a typo in the first sentence of the Neocortex sub-section of the Materials and Methods section.

The concentration should instead state ‘100 µmol/L’ instead of ‘100 mmol/L’

