# Supplementary material for: Border-associated macrophages promote cerebral amyloid angiopathy and cognitive impairment through vascular oxidative stress
Source: Mol Neurodegener. 2023 Oct 3;18:73. doi: 10.1186/s13024-023-00660-1 (PMC10548599; doi:10.1186/s13024-023-00660-1)
Supplement: Supplementary file 1 — Additional file 1. [file 13024_2023_660_MOESM1_ESM.docx]

**Additional Files**


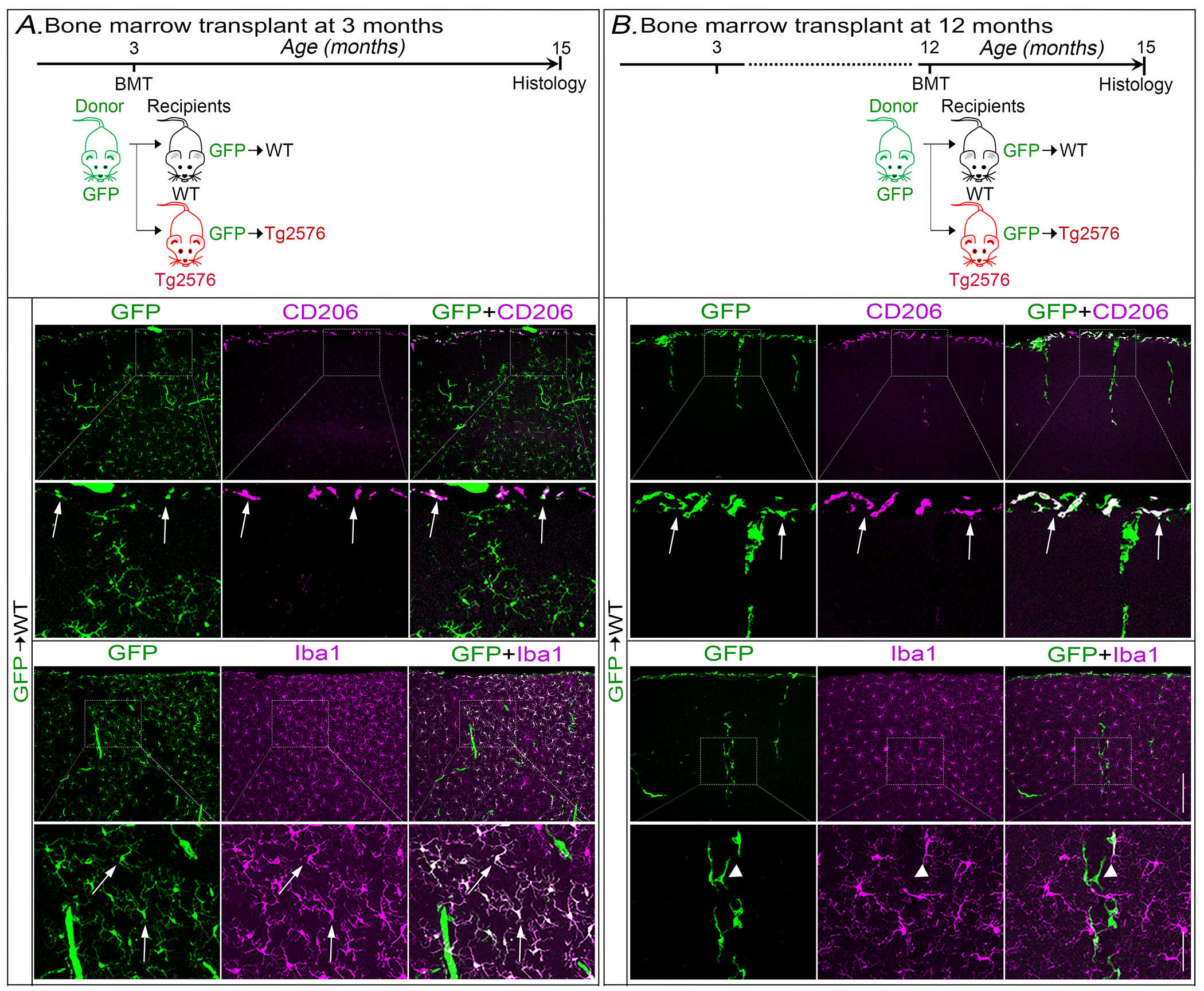


**Fig. S1:** Outcome of GFP^+^ BM transplantation at 3 or 12 months of age followed by identification of GFP^+^ cells at 15 months of age. This figure pertains to GFP^+^ BM transplant in WT mice. See Fig. 1 for GFP^+^ transplantation in Tg2576 mice. ***A:*** GFP^+^ BM was transplanted into WT littermates at 3 months and GFP^+^ cell identity determined at 15 months of age. GFP^+^ cells are seen surrounding cerebral blood vessels, which are also positive for the BAM marker CD206, as well in the parenchyma, which have the morphology of microglia and are strongly Iba1^+^. ***B****:* GFP^+^ BM was transplanted in WT littermates at 12 months and GFP^+^ cell identity determined at 15 months of age. GFP^+^/CD206^+^ cells are seen surrounding cerebral blood vessels, no GFP^+^/Iba1^+^ were observed in the brain parenchyma. In ***A,B***, arrows indicate co-localization and arrowheads no co-localization. N=4-5/group; Two-way ANOVA with Tukey’s test; mean±SEM; scale bars in ***A*** and ***B***, 200 and 50 µm; data presented as mean±SEM.


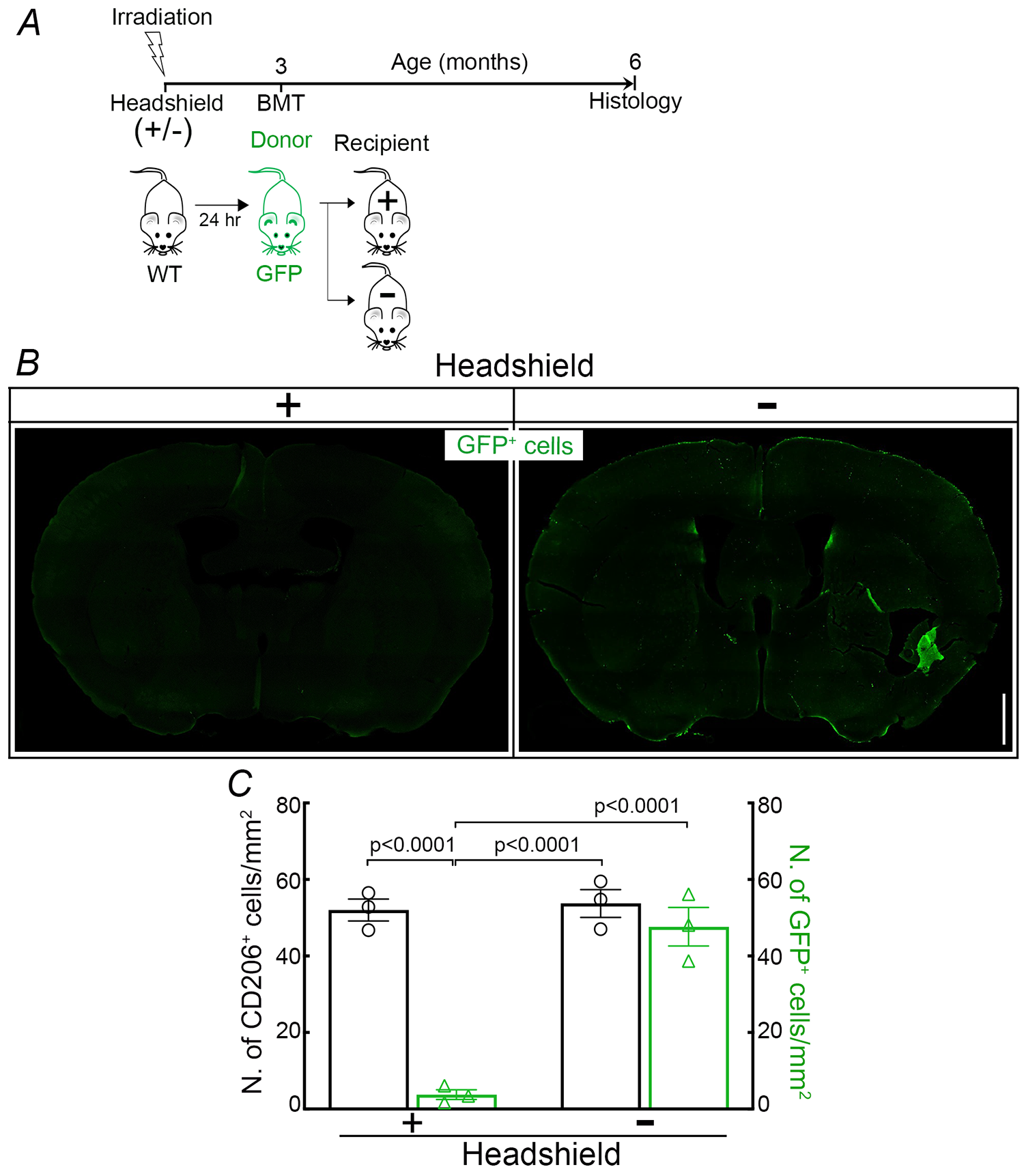


**Fig. S2.** Effect of head shielding during irradiation for GFP^+^ BM transplant on entry of GFP^+^ cells into the brain. ***A*:** Cartoon depicting the experimental protocol. ***B****:* Representative whole-brain section for GFP^+^ cells with (+) or without (-) headshield. Scale bar: 1 mm. ***C*.** Quantification for GFP^+^ cells and CD206^+^ cells. N=3/group; data were analyzed by two-way ANOVA with Tukey’s test; data presented as mean±SEM.


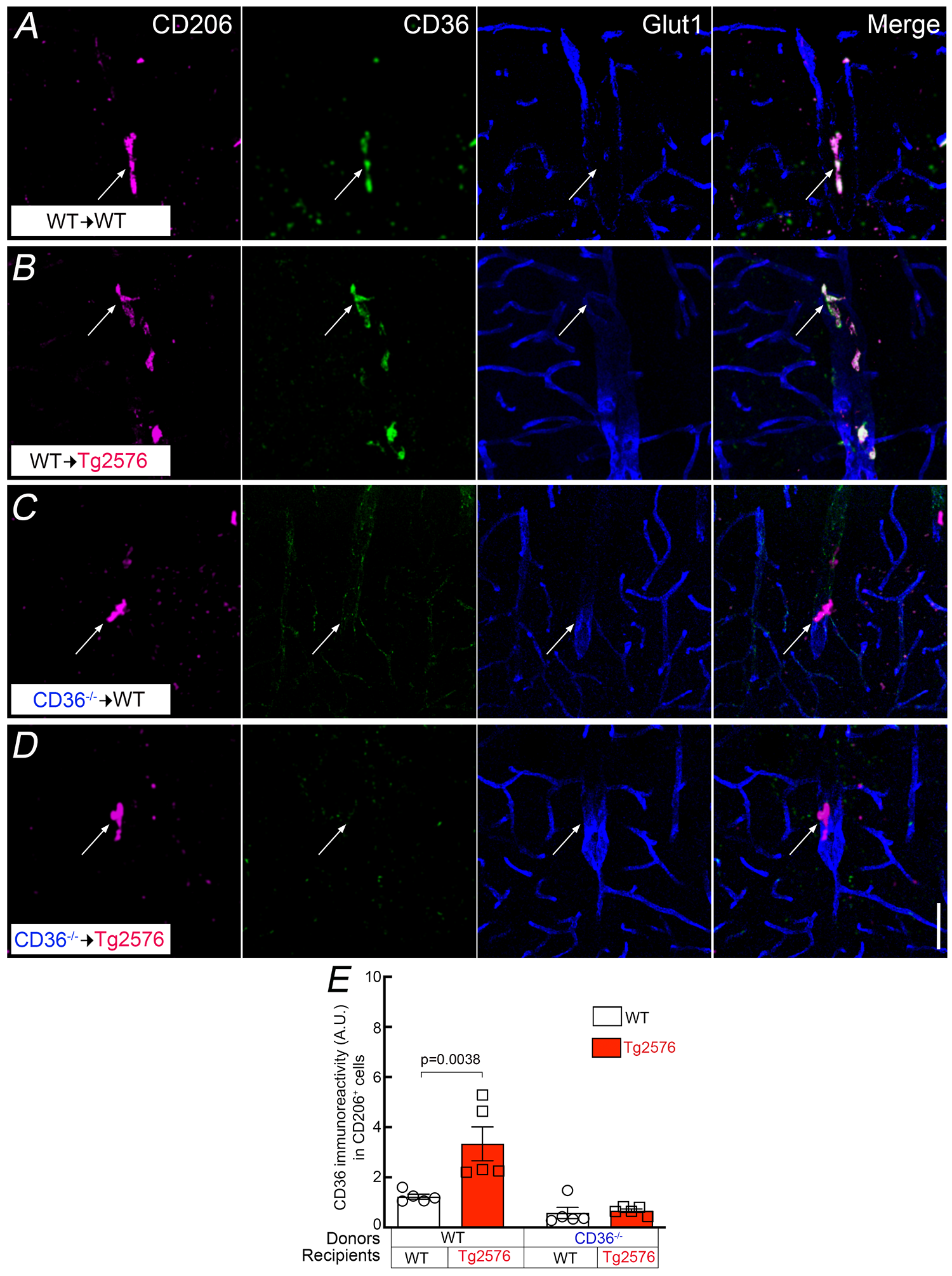


**Fig. S3.** CD36 expression in BAM in 15-month-old WT and Tg2576 mice receiving WT or CD36^-/-^ BM. ***A, B*:** In WT→WT and WT→Tg2576 chimeras CD206^+^ cells (BAM) also express CD36 and are closely associated with Glut-1^+^ blood vessels. ***C,D:*** In CD36^-/-^ →WT and CD36^-/-^ →Tg2576 chimeras CD206+ cells do not express CD36. Scale bar: 50 µm. **E:** Quantification of CD36 immunoreactivity in CD206^+^ cells in the chimeras. N=5/group; two-way ANOVA with Tukey’s test. Data presented as mean±SEM.


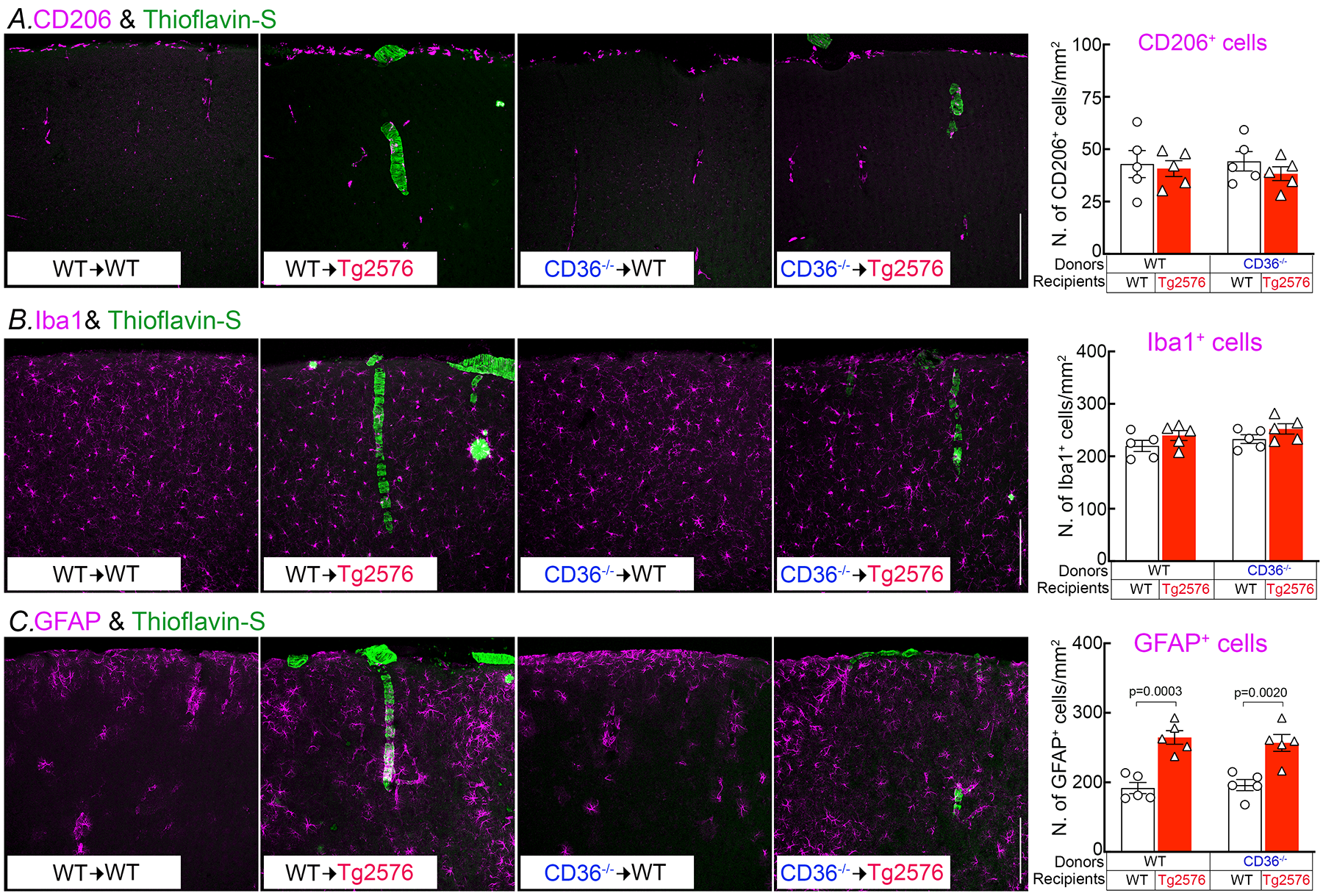


**Fig. S4.** Effect of CD36^-/-^ BM transplantation on CD206^+^, Iba1^+^, and GFAP^+^ cells in 15-month-old WT and TG2576 mice. Coronal cortical sections were co-stained with thioflavin-S to detect amyloid pathology. ***A, B, C:*** CD36^-/-^ BM transplantation in WT and Tg2576 mice does not affect the number of CD206^+^, Iba1^+^ and GFAP^+^ cells. N=5/group; two-way ANOVA with Tukey’s test; scale bar: 200 µm. Data presented as mean±SEM.
